# Neurotrauma: The Crosstalk between Neurotrophins and Inflammation in the Acutely Injured Brain

**DOI:** 10.3390/ijms18051082

**Published:** 2017-05-18

**Authors:** Lindolfo da Silva Meirelles, Daniel Simon, Andrea Regner

**Affiliations:** Programa de Pós-Graduação em Biologia Celular e Molecular Aplicada à Saúde, Universidade Luterana do Brasil (ULBRA), Canoas CEP 92425-900, Brazil; lindolfomeirelles@gmail.com (L.d.S.M.); danielsimon@uol.com.br (D.S.)

**Keywords:** traumatic brain injury, neurotrophins, acute neural injury, inflammation, neuroprotection, traumatic penumbra, brain derived neurotrophic factor, neurovascular unit, pericytes

## Abstract

Traumatic brain injury (TBI) is a major cause of morbidity and mortality among young individuals worldwide. Understanding the pathophysiology of neurotrauma is crucial for the development of more effective therapeutic strategies. After the trauma occurs, immediate neurologic damage is produced by the traumatic forces; this primary injury triggers a secondary wave of biochemical cascades together with metabolic and cellular changes, called secondary neural injury. In the scenario of the acutely injured brain, the ongoing secondary injury results in ischemia and edema culminating in an uncontrollable increase in intracranial pressure. These areas of secondary injury progression, or areas of “traumatic penumbra”, represent crucial targets for therapeutic interventions. Neurotrophins are a class of signaling molecules that promote survival and/or maintenance of neurons. They also stimulate axonal growth, synaptic plasticity, and neurotransmitter synthesis and release. Therefore, this review focuses on the role of neurotrophins in the acute post-injury response. Here, we discuss possible endogenous neuroprotective mechanisms of neurotrophins in the prevailing environment surrounding the injured areas, and highlight the crosstalk between neurotrophins and inflammation with focus on neurovascular unit cells, particularly pericytes. The perspective is that neurotrophins may represent promising targets for research on neuroprotective and neurorestorative processes in the short-term following TBI.

## 1. What Happens to the Acutely Traumatized Neural Tissue?

Worldwide, traumatic brain injury (TBI) is the leading cause of death and severe disability in young adults; its incidence is increasing in the elderly and in the developing world [1,2,3,4]. Based on the Glasgow Coma Scale (GCS), TBI is classified into mild (GCS scores 14–15), moderate (9–13), and severe (3–8) [5]. Severe TBI remains with an outstanding mortality because up to 50% of the patients will still die and nearly all survivors will present some degree of sequelae in spite of the advances in trauma care [6,7,8,9,10,11]. In fact, regardless of over dozens of phase III clinical trials, there are currently no specific treatments known to improve TBI outcomes [12]. Among the most studied predictors of outcome after severe TBI, age is a consistent predictor, as well as GCS scores and pupillary parameters [4]. Moreover, recent studies show a series of blood biomarkers that are useful in the clinical status evaluation of these patients, such as protein S100B and neuron-specific enolase [13,14,15,16] among a number of promising new candidates [13,14,17,18,19,20,21,22,23]. Thus, establishing the real burden of TBI remains challenging because of its heterogeneity in terms of pathobiology, clinical presentation, and outcome (mortality rates range from less than 1% in mild TBI to up to 50% in severe TBI) [24,25,26].

### 1.1. Traumatic Penumbra

The brain is a highly active metabolic and complex organ of our body and disruption in its normal functioning can lead to loss of homeostasis having devastating implication on the whole body. TBI is unique since it is acquired from an external force. Primary injury involves tissue damage caused directly by the mechanical forces of impact that produce deformation of the brain tissue [1,2]. These forces directly damage the neurons, axons, dendrites, glia, and blood vessels in a focal, multifocal, or diffuse pattern [27]. The immediate damage produced by these primary traumatic forces is usually not alterable and triggers a secondary wave of biochemical cascades, together with metabolic and cellular changes, occurring within seconds to minutes after the trauma and lasting for days, months, or years [27]. This progressive brain damage is characteristic of secondary injury and may culminate in important neural cell death [27,28,29]. Typically, initial neuronal death following acute brain injury occurs by necrosis, on a time scale of minutes, then, a second wave of delayed cell death occurs mostly by apoptosis [12,30,31,32].

Together, neurons, associated glia, and cells of the blood–brain barrier (BBB) form the “neurovascular unit” (NVU). The NVU is at the basis of neurovascular coupling, which allows cerebral blood flow to local regulation according to neuronal activity in specific areas of the brain. TBI may damage the entire NVU as a result of either primary or secondary injury processes [14]. Notably, in the setting of TBI, transfer of energy due to physical impact may cause mechanical deformation of the BBB endothelium [33,34], compromising barrier integrity. The dysautoregulation of brain vessels and BBB disruption leads to the development of brain edema, increased intracranial pressure (ICP), and finally, decreased cerebral perfusion [35]. Accordingly, as brain volume begins to increase due to edema, compensatory mechanisms are exceeded, ICP increases exponentially, and correlates with increased mortality and poor functional outcomes, particularly in the first 96 h after TBI [14,27,36,37]. Increases in ICP, give rise to either focal or global ischemia, leading to further edema and ultimately irreversible brain injury [36,38]. Reduced blood flow and oxygen metabolism in the brain promote a metabolic switch from the aerobic process to an anaerobic program. Metabolic changes occur following TBI of all severities; with regional, multifocal, and/or global abnormalities in metabolism likely occurring as a consequence of secondary neural injury progression [39,40,41]. In this scenario of metabolic crisis, astrocytes may exert a neuroprotective action supplying substrates of glycogen metabolism for the survival of ischemic neurons and oligodendroglial cells. In addition, astrocytes synthesize and release different protective molecules for neurons [42]. Nevertheless, swollen and ischemic neurons and swollen perineuronal astrocytes are considered an “abnormal metabolic cooperation” of the astrocyte-neuronal unit and, indeed, present altered response to chemical signals [43,44].

Following TBI, brain lesions are not limited to the site of the primary trauma, but expand progressively and centrifugally. Therefore, secondary brain injury develops and progresses in the “traumatic penumbra”, that is the potentially salvageable brain tissue surrounding the primary lesion [45,46]. There is evidence that focal necrosis increases over time and the volume of necrotic tissue can reach 400% of the initial lesion 24 h after impact [47]. Clinical studies have also demonstrated that expansion of the penumbra impairs cerebral blood flow and leads to edema and compromised local metabolism, resulting in clinical deterioration [48,49,50]. Pathophysiologically, the “traumatic penumbra” involves a series of damage cascades that give rise to neuronal apoptosis [51,52]. Consequently, ongoing secondary injury occurs in areas of “traumatic penumbra”, where neural tissue struggles for survival. While, on the one hand, cellular cascades culminate in cell death, on the other hand, cell injury triggers mechanisms for neuroprotection and cell survival. In this review, we will present an overview of cellular phenomena involved in the “traumatic penumbra”; particularly, excitoxicity, oxidative stress, mitochondrial dysfunction, and neuroinflammation, processes that contribute to neurological deficits separately, but, at the same time, interact, worsening the progressive outcome of TBI [41,53,54,55].

### 1.2. Mechanisms Involved in Ongoing Secondary Injury Shortly After Traumatic Brain Injury (TBI)

In the injured brain, excitotoxicity derives from an acute increase in extracellular glutamate levels due to excessive release from presynaptic nerve terminals of depolarized neurons; leakage from neuronal and glial (mainly astrocytic) cells exhibiting damaged/perturbed membranes; or the extravasation through a disrupted BBB [41,53,54,55]. Pathogenesis of TBI also involves enhanced glutamatergic activity at extrasynaptic sites due to failure of glutamate uptake, gliotransmission, reverse operation of the glutamate transporters, increase in presynaptic glutamate release or increase in the number and/or stability of *N*-methyl-d-aspartate-receptors, a subtype of glutamate receptors [41,53,56,57]. The increase in glutamate levels occurs several minutes after the primary trauma, peaks in about 10 min, and stays increased for several days [55]. A crucial event that triggers metabolic dysfunction is the increased release of glutamate into the extracellular milieu following injury, causing marked increases in cerebral glucose use and accumulation of extracellular lactate [41,58,59,60,61,62,63]. Deregulated cerebral metabolism leads to a deficit in cerebral energy production. Subsequently, reductions in ATP cause the failure of ATP-dependent ion channels and proteins leading to ionic osmotic alterations that result in cell swelling and culminates in cell death [64].

Moreover, excitotoxicity causes calcium influx and overload [65,66], resulting in cellular damage due to several mechanisms (i.e., activation of destructive calcium-dependent proteases, oxidative stress, mitochondrial impairment and transition pore formation, and apoptotic events) [39,41,53,67,68,69]. Notably, the production of reactive oxygen species (ROS) is enhanced by TBI [70,71,72]. The high rate of oxidative metabolism and elevated levels of polyunsaturated lipids render the brain especially vulnerable to oxidative stress [73], nevertheless, under physiological conditions, numerous endogenous antioxidants prevent oxidative damage (i.e., superoxide dismutase, glutathione peroxidase, catalase, and low-molecular-weight antioxidants) [74]. However, in the setting of TBI, those neuroprotective systems become overwhelmed, and result in oxidative cell damage. Overproduction of ROS in the mitochondria is an early event that precedes the release of pro-apoptotic factors and activation of caspases, which promote apoptosis as well as necrosis [75]. Specifically, influx of calcium causes production of ROS in the mitochondria, leading to its swelling and compromised function instigating impaired energy metabolism [39,76]. Therefore, the repair of damaged tissue needs more energy than its normal physiological condition. This results in what has been termed a “flow/metabolism mismatch”, which is an important unfavorable factor in secondary injury progression of “traumatic penumbra” after TBI [53,77].

### 1.3. Neuroinflammation Shortly after Traumatic Brain Injury (TBI)

The brain has been traditionally considered a site of “immunologic privilege” because of the lack of lymphatic system and the presence of a relatively impermeable BBB to activated immune/inflammatory cells, but now neuroinflammation is recognized as prominent in the short- and long-term consequences of neural injuries after TBI [78]. The inflammatory response of the brain to TBI is multifactorial, encompassing the activation of resident central nervous system (CNS) immune cells and the cerebral infiltration of peripheral immune cells (through a disrupted BBB), both of which mediate inflammatory processes through a variety inflammatory cytokines, chemokines, adhesion molecules, ROS, and complement factors, among others [79,80]. In response to perturbations in tissue homeostasis, local activation of microglia occurs [81] mediating inflammation through the production of various cytokines, proteases, and ROS [73]. The microenviroment in which microglia are activated influences their phenotype. An M1 phenotype, also referred to as “classical” activation, is promoted in the presence of lipopolysaccharide and interferon-γ (IFN-γ) [82,83], and is characterized by the increased synthesis of pro-inflammatory cytokines and low levels of anti-inflammatory cytokines. Conversely, when exposed to IL-4 or IL-13, microglia polarize into the M2 phenotype [84], and this “alternative activation” reduces the inflammatory response and increases the production of IL-10, transforming growth factor 1β (TGFβ) (both anti-inflammatory), and suppressor of cytokine signaling [83]. When appropriately queued, microglia can also release neurotrophins to augment neuronal growth and survival [85]. Deficits in the ability of microglia to perform these functions or to appropriately switch between M1 and M2 phenotypes detrimentally affect brain function [86]. Microglial activation within the injured area is observed within 6 to 48 h post-injury [73] but evidence has shown that microglia can maintain a primed, or pro-inflammatory profile for weeks to months after the acute effects of injury have dissipated [87]. Astrocytes also play a role in the “traumatic penumbra”, up-regulating neurotrophic factors, increasing cell proliferation, and promoting the long-term survival of neurons by inhibiting apoptosis [87]. However, when astrocytes become over activated, they may form a glial scar, which encapsulates and isolates the axons. While the glial scar may protect the circumjacent healthy tissue from the neurotoxic environment of the “traumatic penumbra”, it may also impair neuroregeneration of the injured tissue [86,88].

In post-TBI, there is an increased neutrophilic and macrophages/monocytes infiltration, astrocytosis, edema, and both pro- and anti-inflammatory cytokines [43,89,90]. Overall, alterations in regional, intrathecal, and systemic concentrations of several inflammatory cytokines (interleukin (IL) -1, -1β, -6, -8, -10, and -12, and tumor necrosis factor α (TNFα) have been observed following human and experimental TBI [19,91,92,93,94,95,96]. Depending on their concentrations and the timing/conditions of their expression following TBI, the interleukin-family may also have beneficial roles in the injured brain, possibly setting the stage for and promoting regenerative and reparative processes [41]. The dual role of the interleukin-family shows a pro-inflammatory phase in the first hours and days after TBI followed by a reparative phase lasting for days to months [97]. Importantly, IL-1β acts uniquely on astrocytes, that when damaged stimulate the release of matrix metalloproteinases (MMPs) [98]. MMPs degrade extracellular matrix and further cause BBB breakdown promoting and prolonging neuroinflammation [99]. Overall, in the acute stage of TBI, neuroinflammation mobilizes immune cells, astrocytes, cytokines, and chemokines toward the “traumatic penumbra” to promote an anti-inflammatory response against neural injury progression, while, latter, excessive activation of inflammation leads to an “inflamed” brain [30,88,100]. Additionally, peripheral injuries of the multi-injured patient may further increase circulating levels of many of the inflammatory cytokines worsening TBI outcomes [12,66].

## 2. What Are the Roles of Neurotrophins after Acute Neural Injury?

Notwithstanding the previous characterization of the pathophysiologic responses to TBI, these biologic responses occur in individuals who possess biologic differences that can modify their response to injury [41,101]. Over the last years, evidence has showed that the brain is capable of significant structural and functional repair, plasticity and regeneration. Approaches for accomplishing this include reawakening the growth potential of the surviving neurons or antagonizing the inhibition of axonal growth and synaptogenesis. Alternatively, cellular replacement is achievable in certain brain regions that possess nascent neural stem cells [25,31,102,103,104]. Thus, the discussed concept of “traumatic penumbra” imbues the transition between injury and repair at the NVU with profound implications for selecting the appropriate type and timing of neuroprotective interventions. Noteworthy, neural tissue disruption and penumbra areas trigger mechanisms for neuroprotection and cell survival. In this scenario, the role of neurotrophins in the “traumatic penumbra” reveals a promising field for research [105,106,107,108,109,110]. Collectively, the discussed evidence raises major questions addressed in the following topics of this review. In the short-term after acute neural injury due to moderate/severe TBI, what cellular pathways could be involved in the recruitment of signals for regeneration or exacerbation of injury? Furthermore, what phenomena can make individual response to neural injury so variable and thus difficult to predict in the first days after trauma? Could, in this scenario, neurotrophins be molecules with key roles in neuroprotection and neurorestoration? In the sections below, we focus on the role of neurotrophins in the acute post-injury response. We also discuss possible endogenous neuroprotective mechanisms of neurotrophins in the traumatic penumbra, while highlighting the crosstalk between neurotrophins and inflammation with focus on pericytes in the neurovascular unit.

### 2.1. Neurotrophins

The brain preserves a capacity to recover and adapt secondary compensatory mechanisms when tissue is compromised. This capability is due to neuroplasticity, a unique feature that makes the neural circuits malleable and is at the basis of memory formation and learning as well as in adapting to injuries and traumatic events throughout life [111]. Neuroplasticity, in the context of TBI, is dependent on pathophysiologic conditions. Specifically, in areas of “traumatic penumbra”, neurotrophins may offer protection from a secondary injury by stimulating growth and differentiation, and promoting recovery of injured brain neurons [112].

Neurotrophins are a family of proteins that includes four structurally related factors: brain-derived neurotrophic factor (BDNF), nerve growth factor (NGF), neurotrophin-3 (NT-3), and neurotrpohin-4 (NT-4)—that regulate a wide variety of neural functions. Neurotrophins act through two distinct receptors: a high-affinity and selective receptor tyrosine kinase family called Trk (containing three members: TrkA, TrkB, and TrkC) and a low affinity receptor called p75, which is a member of the TNFα receptor superfamily [113]. Whereas each neurotrophin has a preferred Trk (NGF binds TrkA, BDNF and NT-4 bind TrkB, and NT-3 prefers TrkC but also binds TrkA and TrkB), all neurotrophins bind p75 with a similar affinity (usually low) [114]. Neurotrophins are initially synthesized as precursors or proneurotrophins. While the Trk receptors bind preferentially to mature neurotrophins, the p75 receptor can bind and be activated by both proneurotrophins and mature neurotrophins. However, Trk receptors alone cannot discriminate between neurotrophins; both Trk and p75 are necessary to confer high-affinity binding and ligand specificity to the neurotrophins, and to influence most actions of neurotrophins on neuronal survival and differentiation [115]. Moreover, the presence of co-receptors in the plasma membrane can alter the cellular response; for instance, p75 alone potentiates TrkA survival pathways and, in association with sortilin, induces cell death [116].

Pro- and mature neurotrophins often elicit opposite effects given their differences in receptor binding [117]. Studies with animal TBI models suggested that some of the pathological and behavioral effects of TBI might be due to damage signaling activated by pro-neurotrophin/p75 association, which prevails over the protective signaling activated by mature neurotrophin/Trk binding [118]. Trk-signals are normally required for neuronal maintenance and function, and defects in Trk activation are associated with early stages of neurodegeneration [119,120,121]. The p75 receptors are implicated in normal developmental pruning and neuronal death. When upregulated and activated in certain tissues, they can cause neurotoxicity associated with neurodegenerative diseases [122,123].

### 2.2. Roles of Neurotrophins Shortly after TBI

BDNF regulates cell proliferation and survival, neuronal plasticity and neuronal cell growth, and is also associated to long-term memory regulation [124]. BDNF is by far the most abundant of neurotrophins found in the human brain [125]. Interestingly, studies have shown high levels of BDNF after experimental TBI, suggesting that this protein may play a role on the pathophysiology of this type of trauma. However, the role of BDNF in a TBI scenario remains controversial, with some experimental studies reporting it as being neuroprotective [126,127,128,129,130] and other studies implicating it as either being neurodegenerative [131] or having no effect on recovery [132]. Studies conducted in humans also observed conflicting results. A cohort of children with severe head injury presented high levels of BDNF in cerebrospinal fluid (CSF) up to 24 h; nevertheless, in that study, only NGF levels in the CSF were indicative of a good outcome [133]. Similar results were observed in a study in which patients presented higher levels of BDNF post-TBI versus controls and were associated with time until death [134]. On the contrary, one study showed that adult TBI patients had lower serum BDNF levels than healthy controls, and that lower BDNF values were also associated with incomplete recovery after TBI [135]. Additionally, some studies observed no significant association between TBI outcome and BDNF levels measured in the plasma of adult patients [17] or in CSF of pediatric patients [136]. The discrepancies between the studies mentioned above can be explained in part by the differences between proBDNF and mature BDNF, since most studies do not differentiate between the neuroprotective mature BDNF and the neurodegenerative proBDNF. The proBDNF/p75 association can lead to negative effects such as apoptosis, while BDNF/TrkB association elicits positive functions such as cell survival [137].

NGF plays a crucial role in survival and maintenance of sympathetic and sensory neurons systems and may play an important role in the regulation of the immune system [138]. It has been proposed that changes in NGF levels in the CNS might be a neuroprotective response after TBI. Prospective observational clinical studies, conducted with children, demonstrated that early increase in NGF concentration in CSF was a marker of brain damage following severe TBI, being associated with better neurologic outcomes [136,139,140,141]. Studies with experimental brain injury have shown that exogenous NGF intraventricular administration prevents or significantly reduces severe neurologic deficits, apoptosis, and brain cell death [142]. Although genes and pathways involved in the neuroprotective role remain largely unknown, studies have shown that NGF can influence neurogenesis and neuronal repair [143].

In the scenario of TBI, the role of NT-3 and NT-4 has been evaluated most in experimental studies. NT-3 has been reported to promote post-traumatic neuroprotection, brain recovery, improvements in neurological functions, and attenuate neuronal injury [144,145,146,147,148,149]. In addition, NT-4 administration has been demonstrated to be neuroprotective in a wide variety of experimental TBI models, the NT-4 administration promotes an adaptive neuroprotective response in the injured brain [150,151,152,153,154,155]. Only one study evaluated NT-3 and NT-4 in TBI patients [156]. Increased NT-4 blood concentrations were correlated with severity of the head injury in adult patients, but for NT-3 concentrations a negative correlation was observed [156]. Since experimental studies observed the neuroprotective effect of these neurotrophins, and NT-3 and NT-4 seem to have an important biological role in TBI scenarios, they need to be studied more in humans.

There are no clinical trials regarding the application of neurotrophins in patients with TBI. However, two studies administered NGF in children with hypoxic-ischemic brain injuries and a prolonged comatose state [157,158]. The studies, both with only two cases, showed that intraventricular NGF infusion may be beneficial after brain injury, suggesting that more prospective, long-term, larger studies are needed to help understand the role of neurotrophins as therapeutic agents for TBI.

Besides the studies evaluating neurotrophins as TBI biomarkers, there was an interest about how genetic variability in neurotrophin genes can influence the outcomes after TBI. Genetic aspects may influence both brain susceptibility to injury and the ability for neural renewal and reorganization. Studies have associated genetic factors to the clinical spectrum outcomes that may follow as a result of TBI. Understanding the influence of genetic polymorphisms on TBI outcomes is still preliminary, except for those that analyzed apolipoprotein E (APOE) polymorphisms, with few studies so far published (reviewed in [159]). There are some potential advantages that can arise as a result of this understanding. Clinically, it may be important in the therapeutic trials, for example. Additionally, it may be used in risk stratification protocols, with genetic data helping in prognosis prediction, adding information to conventional data such as clinical history, initial GCS, and image exams [160].

There are several polymorphisms in neurotrophin genes that have been studied in different neurological diseases, but only BDNF polymorphisms were evaluated in the context of severe or moderate TBI. The most studied BDNF polymorphism is Val66Met (rs6265). The presence of the methionine allele has been associated with less activity-dependent BDNF secretion, as well as abnormal BDNF localization [161]. On the contrary to previous studies that reported that the Met66 allele was associated with relatively impaired cognitive functions in healthy individuals, studies in a military sample of male Vietnam combat veterans with focal penetrating head injuries and non-head-injured normal control group have shown that the Met66 allele was associated with post-injury recovery of general cognitive intelligence [162,163,164].

The role of Val66Met polymorphism in consciousness recovery and cognitive function was studied in patients who were in a vegetative state one month after severe TBI. Scores of levels of cognitive functioning were evaluated retrospectively at 1, 3, 6, and 12 months post-TBI and there were no significant differences found in genotype frequencies between groups who emerged or not from the vegetative state [165].

Studies evaluated the inclusion of data from BDNF polymorphisms in models of prediction of post-TBI mortality. Two BDNF polymorphisms (Val66Met and rs7124442) were studied in severe TBI patients. The results suggested temporally specific prognostic factors for mortality: (a) acutely (0–7 days post-injury), subjects in the group with the hypothesized risk alleles (including Met allele) had the highest survival probability regardless of age; and (b) postacutely (8–365 days), BDNF alleles interacted with age in as much that younger participants with the hypothesized no-risk alleles had the highest survival probability, while older participants with the hypothesized no-risk group had the lowest survival probability [160]. These results suggested that BDNF is a dynamic and genetically modifiable biomarker for mortality and global outcome following TBI.

Collectively, the picture emerging from the studies on effects of neurotrophins in TBI highlights that their effects depend on binding to their receptors, presence of different co-receptors at the plasma membrane, abundance of receptor in specific cell types, and genetic variation, and may be neuroprotective or promote injury progression depending on the complex biochemical and molecular cascades triggered by TBI [166,167].

## 3. What Happens to Neural Tissue during Recovery after Acute Neural Injury?

**Figure 1:**
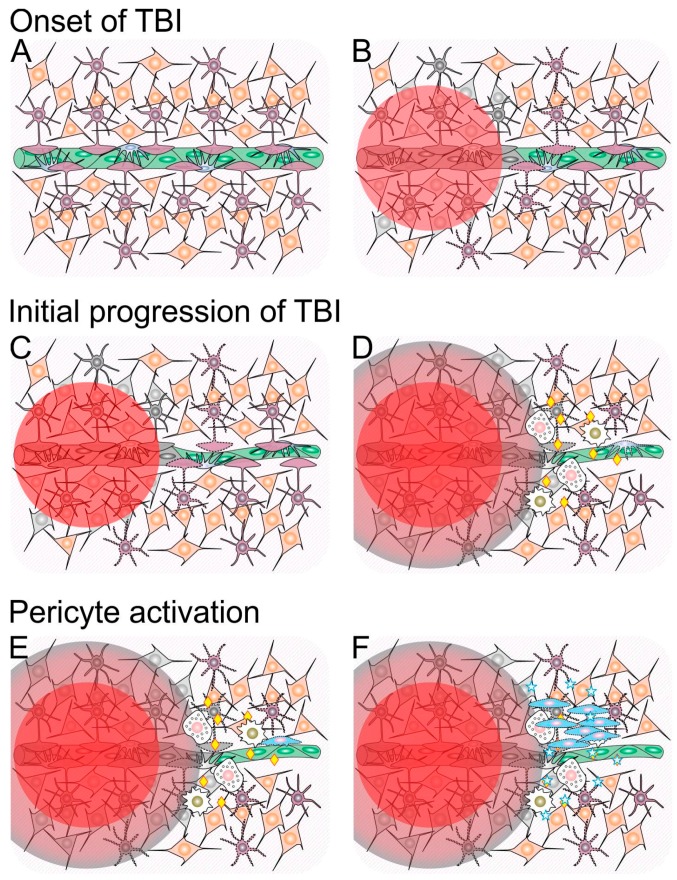
Schematic representation of cellular and molecular events involved in the progression and resolution of traumatic brain injury (TBI). (**A**) Integer neural tissue composed of neurons (orange) and astrocytes (purple) nearby a blood capillary composed of endothelial cells (green) and pericytes (blue). For the sake of simplification, other cell types present in the neural tissue (e.g., oligodendrocytes, microglial cells) and the basement membrane that surrounds the capillary are not shown; (**B**) TBI causes a primary neural injury, represented by a red circle, which leads to cell death (represented by color change to gray); (**C**) Glial cells surrounding the primary lesion site become activated (represented by a change from a solid to a dotted outline), and vasoconstriction takes place; (**D**) Within a few hours following TBI, dying cells in the primary lesion site release a number of toxic molecules that diffuse to the surroundings (penumbra represented in red-to-gray gradient), which causes further cell death and glial cell activation. Concomitantly, the blood–brain barrier is disrupted (represented by loss of coverage of the capillary by astrocyte feet), and innate immune system cells (white), including monocytes/macrophages (golden nuclei) and neutrophils (pink nuclei), reach the site and release a number of inflammatory signaling molecules (yellow diamonds); (**E**) During the first day after TBI, inflammation increases and is accompanied by pericyte activation (represented by a change from a solid to a dotted outline); (**F**) Between one and three days after TBI, activated pericytes leave their native perivascular niche, proliferate and secrete a number of antiapoptotic, trophic and immunomodulatory molecules (represented as blue stars) that counteract cell death and inflammation triggered by TBI.

The events that ensue after TBI are complex, and the outcome may be viewed as a consequence of the manner in which these events take place. To develop this topic and, at the same time, consolidate concepts discussed in the previous sections, Figure 1 provides a schematic timeline of these events. According to this schematic view, the first mechanism that acts to limit cell death in the damaged tissue is glial cell activation (Figure 1B). During the course of various types of neural tissue injury, activated glial cells proliferate and secrete antiapoptotic molecules such as NGF, glial cell-derived neurotrophic factor (GDNF), and NT-3, which contribute to neuron survival [168,169,170]. Activated (i.e., reactive) astrocytes also contribute to decrease the amount of excitotoxic glutamate released by the injured and dead cells [171]. The negative side of this process called reactive astrogliosis is the production of pro-inflammatory molecules, and the formation of a glial scar that limits reestablishment of connections between neurons and compartmentalizes the tissue (reviewed in [172]), which favors edema.

### 3.1. Neuroinflammation and Pericytes

Another significant event during the course of tissue response to TBI is the inflammation that takes place before, during, and following disruption of the BBB as a consequence of the action of microglial cells and infiltrating leukocytes. Since the CNS is devoid of adaptive immune system cells, this inflammation helps prevent possible infections by targeting microorganisms brought in during TBI. Excessive inflammation is detrimental to recovery from neural injury [173]; on the other hand, chemokines released during this process attract monocytes, T cells, and B cells that, aside from possibly triggering an adaptive immune response, may secrete neurotrophins such as BDNF when activated [174], which contributes to neuroprotection. While the participation of immune system cells in neuroinflammation is acknowledged, the contribution of another cell type, namely the pericyte, remains greatly overlooked and is currently becoming recognized. Classically, pericytes have been regarded as important for maintenance of blood vessel integrity [175]. The anatomical position of pericytes, i.e., around and physically connected to endothelial cells, means that cells meant to ingress the tissue must pass not only through the layer of endothelial cells that line the blood vessels, but also through these particular periendothelial cells. In the CNS, pericytes are integral components of the blood–brain barrier (reviewed in [176]).

Some studies have presented evidence that pericytes may contribute to neuroinflammation owing to their ability to perceive infection-related or pro-inflammatory signals, and respond through secretion of chemokines that recruit inflammatory cells [177,178,179]. For example, interleukin 17 secreted by neutrophils has been shown to coax cultured pericytes into secreting pro-inflammatory molecules that, in turn, modulated the neutrophil phenotype [180]. In contrast, cultured pericytes have also been shown to be immunosuppressive, as they can inhibit the proliferation of T cells [181,182]. Even though these data may seem contradictory, it should be noted that studying pericytes is difficult because these cells undergo activation once injury and its associated processes destabilize the tissue where they are; consequently, their phenotype changes as the wound healing process takes place.

### 3.2. The Crosstalk between Neurotrophins and Pericytes

While investigating the origin of the cultured cells operationally called “mesenchymal stem cells” or mesenchymal stromal cells (MSCs), we have proposed that these cells arise from pericytes that become activated in culture [183]. Cultured MSCs are cells able to differentiate along osteogenic, chondrogenic, and adipogenic pathways, and secrete a number of signaling molecules that exert trophic, antiapoptotic, proangiogenic, and immunosuppresive effects (reviewed in [184]). A corollary of the thesis that cultured MSCs arise from pericytes is that, from the onset of tissue injury until resolution of the lesion, pericytes initially contribute to inflammation, but gradually give rise to cells somewhat similar to cultured MSCs, which secrete various bioactive molecules that contribute to wound healing. Owing to these characteristics, these cells have been proposed to be “medicinal signaling cells” [185].

Results from studies that applied controlled cortical impact (CCI) to rats suggest that, after TBI, pericytes play key roles during evolution of the response to the nervous tissue wound. Initially, a number of pericytes die owing to the primary damage and ischemia, while pericytes in the surroundings of the wound promote vasoconstriction (Figure 1C), which further contributes to TBI-induced hypoperfusion [186]. This behavior has been attributed to upregulation of the contractile protein α-smooth muscle actin secondary to upregulation of expression of endothelin receptors A (EDNRA) and B (EDNRB) by pericytes starting at 4–8 h after the injury, with peaks of expression of EDNRB at 8 h, and of EDNRA 24 h after the injury [186]. Earlier data from CCI experiments in rats suggest that, starting at 2 h after injury, some pericytes in areas close to the lesion site start migrating away from capillaries and can be found associated with astrocytes and oligodentrocytes 48 h after injury; whereas, pericytes close to the injury site that fail to migrate may degenerate [187]. Further data from CCI experiments in mice indicate that the number of pericytes decreases during the first 12 h after injury, and that the number of proliferative pericyte-derived cells increases progressively and reaches a peak five days after the injury [188]. In that study, the authors called this process reactive pericytosis, and found that it was delimited by the reactive astrogliosis area around the injury site.

While the above-mentioned data indicate that brain pericytes become activated, migrate, and proliferate in response to TBI, no detailed studies on the bioactive molecules secreted by pericytes or activated pericytes in the CNS are available to confirm they can provide neuroprotection by secretion of antiapoptotic or trophic molecules in situ. Cultured human brain-derived and peripheral nerve-derived pericytes have been shown to express the neurotrophic factors NGF, BDNF, and GDNF [189]. Cultured human brain pericytes were found to express the neurotrophins NGF, BDNF, and NT-3, and expression of the latter was increased under hypoxic culture conditions [190]. In that study, NT-3 was found to upregulate expression of NGF by astrocytes in vitro, which indicates pericytes may boost NGF expression by astrocytes during ischemia or hypoperfusion in the injured neural tissue. Since cultured pericytes exhibit characteristics of activated pericytes, it is possible that activated pericytes provide neuroprotection by means of secretion of neurotrophic factors in the context of brain damage caused by TBI.

The studies on expression of neurotrophins in cultured pericytes do not allow direct inference on the expression of these molecules by pericytes before activation. To try to circumvent the scarcity of information on this topic, we examined microarray expression data of highly purified, non-cultured pericytes isolated from human adipose tissue [191] in search of genes that code for neurotrophic factors. The microarray data processing and analysis procedures used were essentially as described [191]. These results, shown in Table 1, indicate that non-cultured pericytes (ncPCs) express *NGF*, *BDNF*, *NTF3* (which codes for NT-3), a precursor form of GDNF, and *PSPN* (persephin) at appreciable levels. It seems, therefore, that expression of some neurotrophic factors is a characteristic of non-cultured pericytes even if they reside in a non-nervous tissue.

An interesting characteristic of non-cultured pericytes derived from adipose tissue is the expression of p75 (aka nerve growth factor receptor, NGFR, which is encoded by the *NGFR* gene), which has been demonstrated not only at the RNA level (as shown in Table 1) but also at the protein level, on the surface of freshly isolated pericytes [181]. Increased NGF levels early after TBI have been shown to be positively correlated with a positive outcome in pediatric patients [141]. Data from animal models indicate that exogenous administration of NGF [192,193] or molecules that mimic its function as a signaling molecule [194] is beneficial for the outcome of TBI. While signaling through p75 effected by neurotrophins promotes neuroprotection, binding of pro-neurotrophins (precursor forms of neurotrophins) to this receptor may promote cell death, a phenomenon that can be minimized by pharmacological inhibition of the p75 intracellular death domain [195].

Molecular inhibition of pro-NGF binding to p75 after experimental TBI has been shown to improve the outcome in rat TBI models [196]. In a mouse model of heart ischemia-reperfusion injury, expression of pro-NGF increases in cardiomyocytes, while expression of p75 increases in microvascular pericytes, which results in microvascular damage [197]. Therefore, it becomes apparent that, during the course of TBI, the effects of signaling through p75 are relevant not only to neural cells, but also to vascular cells, as binding of pro-neurotrophins produced during this process may undermine vascular supply and the generation of activated pericytes that act as medicinal signaling cells. During this process, endothelial cells are also expected to be compromised as they lose pericyte support. In line with this, we have found that increased levels of the endothelial cell markers von Willebrand factor and matrix metalloprotinase-9 in the blood after TBI are inversely correlated with the outcome [21,198].

As shown above, signaling involving neurotrophins and their receptors influence vascular integrity after TBI, which affects its outcome. Therefore, detection of these molecules along with markers of vascular damage in the blood after TBI may represent a way to monitor the patient’s status and predict his/her outcome. Finally, considering that TBI may yield various outcomes in spite of affecting a rather homogeneous population (young individuals who exhibit good regenerative potential), knowledge on genetic variants the neurotrophins and vascular-associated molecules involved in this process may contribute to understanding the basis of individual variations that underlie these outcomes.

## 4. Conclusions

TBI is a major cause of death and disability in people who otherwise could follow a productive life, which translates into large social and economic burdens to society. Therefore, it is important to adequately assess severity and prognosis of TBI victims as soon as they reach the emergency service. Consequently, knowledge on the factors that underlie TBI progression and outcome is key. Neurovascular unit cells in the penumbra area are important sources of neurotrophins and other signaling molecules that protect neural cells from apoptosis caused by secondary injury after TBI. The assessment of levels of neurotrophins in the blood or cerebrospinal fluid has been shown to contribute to the prediction of the outcome of TBI. Since neurovascular unit cells are responsible for synthesis of these neuroprotective molecules, it becomes apparent that detection of markers of vascular integrity in these fluids may provide important information to this end. As a particular neurovascular unit cell, the pericyte becomes activated to provide trophic support to the neural tissue after TBI, investigating markers of pericyte activation in TBI victims may also provide useful information to predict its outcome.

## Figures and Tables

**Table 1:** Expression message for neurotrophic factors and p75 in pericytes and other cell types. Publicly available microarray expression data available at the Gene Expression Omnibus (GEO) were analyzed. Expression data from non-cultured pericytes (ncPCs; accession GSE71535) were compared with data from cultured pericytes (cPCs) expanded in pericyte medium (PC medium) or mesenchymal stromal cell (MSC) medium (accessions GSM1655126, GSM1655127, GSM1655128, GSM1655129, GSM1655130, and GSM1655131), adipose tissue-derived MSCs (ATMSCs (Adipose Tissue-Derived MSCs); accessions GSM1655122, GSM1655123, and GSM1655124), human umbilical vein endothelial cells (HUVECs (Human Umbilical Vein Endothelial Cells); accessions GSM418611, GSM418615, and GSM418619), and peripheral blood white blood cells (PBWBCs (Peripheral Blood White Blood Cells); GSM469524, GSM469528, GSM469532, GSM469536, and GSM469540). Numbers in the table’s cells represent fluorescence intensity in the microarray spot corresponding to the specified probe after quantile normalization and log2 transformation, which is proportional to expression of the specified transcripts. Cells with expression values were colored according to expression intensity as shown in the scale bar. Note that three different probes (and, consequently, spots) for *BDNF*, and three different probes for *GDNF* were present in the microarrays analyzed. In the case of *GDNF*, two of the probes identify its transcript variant 1, and one of the probes identifies a transcript for a precursor form of GDNF.

Probe Name	Gene Symbol	Description	ncPCs	cPCs (PC Medium)	cPCs (MSC Medium)	ATMSCs	HUVECs	PBWBCs
A_23_P115190	*NGF*	nerve growth factor (β polypeptide)	7.721	4.458	6.394	7.998	3.967	4.790
A_23_P127891	*BDNF*	brain-derived neurotrophic factor, transcript variant 1	6.635	7.903	11.047	8.457	6.650	2.812
A_32_P7316	*BDNF*	brain-derived neurotrophic factor, transcript variant 1	2.739	2.975	8.517	5.321	4.026	2.572
A_23_P127891	*BDNF*	brain-derived neurotrophic factor, transcript variant 1	6.635	7.903	11.047	8.457	6.650	2.812
A_23_P360797	*NTF3*	neurotrophin 3, transcript variant 2	8.034	3.479	3.916	8.211	4.513	3.945
A_23_P4899	*NTF4*	neurotrophin 4 (NTF4)	2.052	2.046	5.979	2.293	2.266	2.352
A_24_P25544	*GDNF*	glial cell derived neurotrophic factor, transcript variant 1	2.277	2.294	4.050	2.979	1.758	1.948
A_23_P167683	*GDNF*	glial cell derived neurotrophic factor, transcript variant 1	4.375	3.359	4.955	2.883	1.868	1.852
A_32_P377880	*GDNF*	glial cell derived neurotrophic factor, precursor	7.660	7.685	9.894	6.382	1.790	2.378
A_23_P90359	*NRTN*	Neurturin	2.839	2.461	4.375	2.853	2.636	3.578
A_23_P410507	*PSPN*	Persephin	8.082	7.490	6.954	6.349	6.620	8.475
A_23_P389897	NGFR	nerve growth factor receptor (p75)	10.493	2.299	3.673	5.042	2.448	3.644
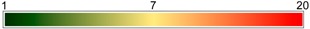								

## Abbreviations

APOE	Apolipoprotein E
ATMSCs	Adipose Tissue-Derived MSCs
BBB	Blood Brain Barrier
BDNF	Brain-Derived Neurotrophic Factor
CCI	Controlled Cortical Impact
cPCs	Cultured Pericytes
CSF	Cerebrospinal Fluid
CNS	Central Nervous System
EDNRA	Endothelin Receptor A
EDNRB	Endothelin Receptor B
GCS	Glasgow Coma Scale
GDNF	Glial Cell-Derived Neurotrophic Factor
GEO	Gene Expression Omnibus
HUVECs	Human Umbilical Vein Endothelial Cells
ICP	Intracranial Pressure
IFN-λ	Interferon-λ
IL	Interleukin
MSC	Mesenchymal Stromal Cell
MMP	Matrix Metalloproteinase
ncPCs	non-cultured Pericytes
NT-3	Neurotrophin-3
NT-4	Neurotrophin-4
NGF	Nerve Growth Factor
NVU	Neurovascular Unit
PBWBCs	Peripheral Blood White Blood Cells
PSPN	Persephin
ROS	Reactive Oxygen Species
TBI	Traumatic Brain Injury
TGFβ	Transforming Growth Factor 1β
TNFα	Tumor Necrosis Factor α
